# A Segmental Copy Number Loss of the *SFMBT1* Gene Is a Genetic Risk for Shunt-Responsive, Idiopathic Normal Pressure Hydrocephalus (iNPH): A Case-Control Study

**DOI:** 10.1371/journal.pone.0166615

**Published:** 2016-11-18

**Authors:** Hidenori Sato, Yoshimi Takahashi, Luna Kimihira, Chifumi Iseki, Hajime Kato, Yuya Suzuki, Ryosuke Igari, Hiroyasu Sato, Shingo Koyama, Shigeki Arawaka, Toru Kawanami, Masakazu Miyajima, Naoyuki Samejima, Shinya Sato, Masahiro Kameda, Shinya Yamada, Daisuke Kita, Mitsunobu Kaijima, Isao Date, Yukihiko Sonoda, Takamasa Kayama, Nobumasa Kuwana, Hajime Arai, Takeo Kato

**Affiliations:** 1 Department of Neurology, Hematology, Metabolism, Endocrinology and Diabetology, Yamagata University Faculty of Medicine, Yamagata, Japan; 2 Genomic Information Analysis Unit, Department of Genomic Cohort Research, Yamagata University Faculty of Medicine, Yamagata, Japan; 3 Department of Neurosurgery, Graduate School of Medicine, Juntendo University, Tokyo, Japan; 4 Department of Neurosurgery, Tokyo Kyosai Hospital, Tokyo, Japan; 5 Department of Neurosurgery, Yamagata University Faculty of Medicine, Yamagata, Japan; 6 Department of Neurological Surgery, Okayama University Graduate School of Medicine, Dentistry and Pharmaceutical Sciences, Okayama, Japan; 7 Department of Neurosurgery, Toshiba Rinkan Hospital, Sagamihara, Japan; 8 Department of Neurosurgery, Noto General Hospital, Nanao, Japan; 9 Department of Neurosurgery, Megumino Hospital, Eniwa, Japan; Sant Joan de Déu Children's Hospital, SPAIN

## Abstract

Little is known about genetic risk factors for idiopathic normal pressure hydrocephalus (iNPH). We examined whether a copy number loss in intron 2 of the *SFMBT1* gene could be a genetic risk for shunt-responsive, definite iNPH. Quantitative and digital PCR analyses revealed that 26.0% of shunt-responsive definite iNPH patients (n = 50) had such a genetic change, as compared with 4.2% of the healthy elderly (n = 191) (OR = 7.94, 95%CI: 2.82–23.79, *p* = 1.8 x 10^−5^) and 6.3% of patients with Parkinson’s disease (n = 32) (OR = 5.18, 95%CI: 1.1–50.8, *p* = 0.038). The present study demonstrates that a copy number loss within intron 2 of the *SFMBT1* gene may be a genetic risk factor for shunt-responsive definite iNPH.

## Introduction

Idiopathic normal pressure hydrocephalus (iNPH) is a disease of the elderly characterized by ventricular enlargement of the brain and symptoms of gait disturbance, cognitive impairment, and urinary incontinence [1,2]. iNPH is clinically important as a treatable gait disturbance and/or preventable dementia by shunt operation [3]. It usually occurs with a sporadic onset; however, genetic factors are suggested to be involved in the pathogenesis of iNPH because of the presence of familial onset (two or more patients with NPH in a single family) of the disease, the clinical and brain MRI features of which are indistinguishable from those of sporadic iNPH [4–8]. The familial aggregation of iNPH has also been reported [7]. Previously, we made a whole-genome analysis for copy number variations (CNV) in community-dwelling Japanese elderly and found that a segmental copy number loss in the 12-kb region within intron 2 of the *SFMBT1* (*Scm-like with four MBT domains protein 1*) gene was observed in 4 of 8 subjects (50%) with possible iNPH and AVIM (asymptomatic ventriculomegaly with features of iNPH on MRI) [9]; AVIM seems to be a pre-symptomatic state of iNPH [10,11]. On the other hand, the frequency of such a genetic change was very low in healthy controls (only one of the 110 controls: 0.9%) [9]. Even now, however, it remains undetermined whether patients with definite iNPH, whose neurological symptoms are improved after shunt operation, show such a change in the gene. The aim of the present study was to clarify whether a segmental copy number loss in intron 2 of the *SFMBT1* gene would also be a genetic risk factor for shunt-responsive, definite iNPH.

## Subjects and Methods

The diagnosis of iNPH was made in accordance with the Japanese Guidelines for Management of iNPH [3]. The guidelines show three levels of diagnostic certainty of iNPH: “possible,” “probable,” and “definite” iNPH. The diagnosis of definite iNPH was made only when one or more symptoms of the iNPH triad were improved by shunt operation. We used 50 patients with definite iNPH (33 men and 17 women; mean age of 78.2 years, ranging from 65 to 87) diagnosed in accordance with the Japanese Guidelines; in brief, patients were 65 years of age or older, had one or more symptoms of the iNPH triad, and showed ventricular enlargement on brain MRI (Evans index > 0.3) with no cerebrospinal fluid (CSF) abnormalities upon examination. All were shunt-responsive with an improvement of one point or more (favorable outcome) on the modified Rankin Scale and/or iNPH Grading Scale with shunt operation [12,13]. There was no family history of iNPH in any patients. In addition, most of the patients showed the MRI findings of DESH (Disproportionately Enlarged Subarachnoid-space Hydrocephalus): narrowing of the subarachnoid space and cortical sulci at the high convexity of the cerebrum and widening of the Sylvian fissures [14]. Because the subarachnoid space and cortical sulci of the cerebrum become enlarged in Alzheimer’s disease due to atrophy of the cerebral cortex, the DESH findings seems to be useful to differentiate iNPH from Alzheimer’s disease [3]. Other diseases causing gait disturbance and/or cognitive impairment were excluded by extensive examinations, including neurological, neuroimaging, and CSF examinations. Eight were inpatients of our hospital, which is located in the northern part of Japan; the others (n = 42) were from 6 hospitals in the central, southern, or northernmost parts of Japan. As a control (control-1), 99 healthy Japanese elderly were used, all of whom were 70 years of age [9]. Neurological examination or brain MRI found no abnormalities in these subjects. We also employed another population of control subjects (control-2) (n = 92), aged 65 years or older, who had no apparent neurological symptoms [15]. In addition, 32 patients with Parkinson’s disease (PD) were used as a disease control. The diagnostic and inclusion criteria for PD have been described elsewhere [16].

**Fig 1:**
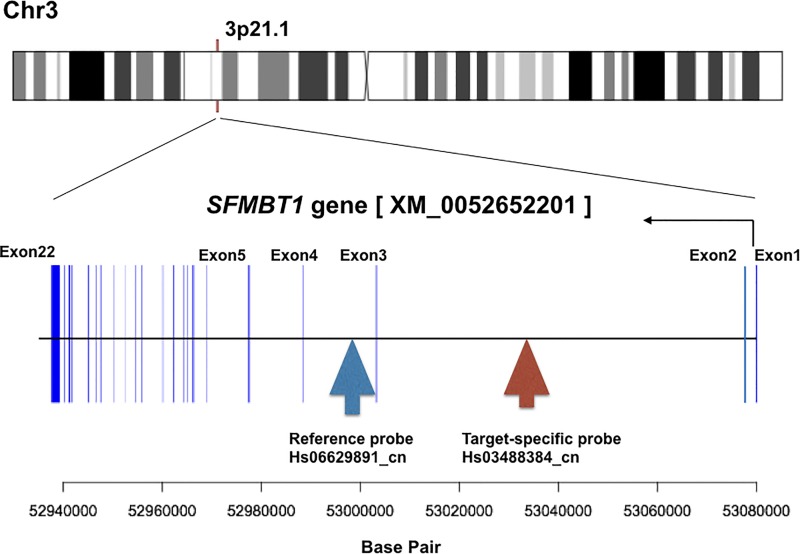
Binding sites of target-specific probe and reference probe. The target-specific probe binds to intron 2 of *SFMBT1* gene (chr3:53035556, NCBI build 37), and the reference probe to its intron 3 (chr3:52995796, NCBI build 37).

Fresh whole-blood samples were obtained from all the cases and controls, and genomic DNA was extracted and purified by the same methods from peripheral leukocytes using a DNeasy Blood & Tissue kit (QIAGEN, Germany). A good quality of DNA was confirmed by using the Agilent 2200 Tapestation system with genomic DNA screen tapes (Agilent Technologies, Workingham, UK). The copy number of the region in intron 2 of the *SFMBT1* gene was measured using real-time quantitative PCR (qPCR) (ABI PRISM 7900, Thermo Fisher Scientific, Inc., USA) and digital PCR (QuantStudio 3D, Thermo Fisher Scientific, Inc.). Two probes of FAM-labeled TaqMan® Copy Number Assays were used: a target-specific probe (pre-designed Hs03488384_cn, Thermo Fisher Scientific, Inc.) was targeted to the region in intron 2 (chr3:53035556, NCBI build 37), and the reference probe (pre-designed Hs06629891_cn, Thermo Fisher Scientific, Inc.) was targeted to its flanking region (chr3:52995796, NCBI build 37) (Fig 1, S1 Fig). A VIC-labeled RNaseP control probe was used for qPCR. In each assay, 10 ng of DNA was used, and PCR reaction was performed in triplicate, in accordance with the manufacturer’s instructions. Copy number change in the region of intron 2 of the *SFMBT1* gene was estimated by the delta-delta CT method in qPCR and from the absolute number of copies/μl in the digital PCR. The concordance rate between the two methods was 82%. The presence of the copy number loss (deletion) was confirmed by a PCR-based amplification of the region with or without deletion in intron 2 (S2 Fig). A statistical analysis was performed using R statistical computing software (version 3.2). Fisher’s exact test was used to test the difference between two categorical variables. *P* < 0.05 was considered to be statistically significant.

All participants gave written informed consent to be included in the present study. The study was approved by the Ethics Committee of Yamagata University Faculty of Medicine and all other institutes and hospitals that participated in the study (the Ethics Committee of Juntendo University Graduate School of Medicine; the Ethics Committee of Tokyo Kyosai Hospital; the Ethics Committee of Okayama University Graduate School of Medicine, Dentistry and Pharmaceutical Sciences; the Ethics Committee of Toshiba Rinkan Hospital; the Ethics Committee of Noto General Hospital; and the Ethics Committee of Megumino Hospital).

## Results

**Fig 2:**
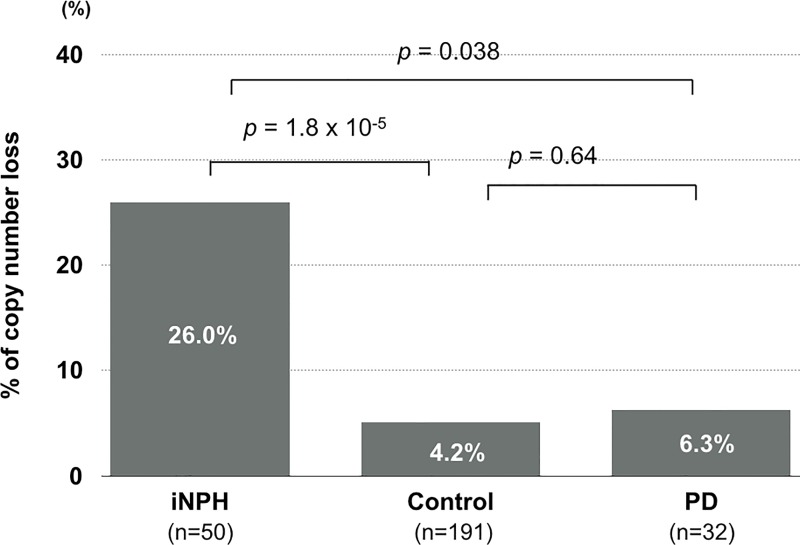
Frequency of copy number loss in intron 2 of *SFMBT1* gene in iNPH, healthy control and Parkinson’s disease (PD).

As a first step, qPCR was used to examine DNA from 8 inpatients with shunt-responsive definite iNPH in our hospital to determine the copy number in the region of intron 2 of the *SFMBT1* gene. The results showed that five of the 8 patients had a heterogeneous copy number loss in the region of intron 2 of the *SFMBT1* gene. Such genetic changes were observed in 5 of the 99 healthy elderly who had no neurological symptoms or brain MRI abnormalities. Next, to test whether the results could be replicated in another population of iNPH, we collected DNA samples of 42 patients with definite iNPH from other (central, southern, and northernmost) areas of Japan and examined the copy number of the region in the *SFMBT1* gene by using digital PCR. The same 99 samples were used as a healthy control. The results showed that the copy number loss in the region of the gene was found in 8 of the 42 patients (OR = 4.37, 95%CI: 1.17–18.22, *p* = 0.021) (Tables 1 and 2). To check the possibility that the control group of healthy elderly we used might not represent the general population of healthy people, we used another independent population of healthy subjects as control-2 (n = 92). The frequency of the copy number loss of the gene was 3.3% in control-2; thus, the above results were again replicated (OR = 6.86, 95%CI: 1.53–42.52, *p* = 0.004) (Tables 1 and 2). To combine and analyze all of the samples together (50 definite iNPH patients vs. 191 controls), the OR was 7.94 (95%CI: 2.82–23.79), and the *p* value was 1.8 x 10^−5^ (Tables 1 and 2, Fig 2).

**Table 1:** Copy numbers of the region in intron 2 of the SFMBT1 gene in iNPH patients and controls.

	Copy number in intron 2 of the *SFMBT1* gene		
	1 (loss)	2 (normal)	3 (gain)
iNPH-1 (n = 8)	5 (62.5%)	2 (25.0%)	1 (12.5%)
iNPH-2 (n = 42)	8 (19.0%)	31 (73.8%)	3 (7.1%)
Total of iNPH-1&2 (n = 50)	13 (26.0%)	33 (66.0%)	4 (8.0%)
Control-1 (n = 99)	5 (5.1%)	91 (91.9%)	3 (3.0%)
Control-2 (n = 92)	3 (3.3%)	88 (95.7%)	1 (1.1%)
Total of control-1&2 (n = 191)	8 (4.2%)	179 (93.7%)	4 (2.1%)
Parkinson's disease (n = 32)	2 (6.3%)	27 (84.3%)	3 (9.4%)

**Table 2:** Frequency of the copy number loss in intron 2 of the SFMBT1 gene in iNPH patients and controls.

**A: iNPH-2 vs. control-1**				
% of copy number loss				
iNPH-2 (n = 42)	Control-1 (n = 99)	Odds Ratio	95% CI	*p* value^a^
19.0%	5.1%	4.37	1.17–18.22	0.021
**B: iNPH-2 vs. control-2**				
% of copy number loss				
iNPH-2 (n = 42)	Control-2 (n = 92)	Odds Ratio	95% CI	*p* value^a^
19.0%	3.3%	6.86	1.53–42.52	0.004
**C: iNPH-1&2 vs. control-1&2**				
% of copy number loss				
iNPH-1&2 (n = 50)	Control-1&2 (n = 191)	Odds Ratio	95% CI	*p* value^a^
26.0%	4.2%	7.94	2.82–23.79	1.8 x 10^−5^

a The difference between two groups was analyzed by Fisher's exact test.

Because gait disturbance, or lower-limb parkinsonism, is a cardinal symptom of iNPH, we analyzed DNA for the copy number loss of the *SFMBT1* gene in 32 patients with PD. The results showed that the frequency of the genetic variant was 6.3% in the PD patients; there was no significant difference in the frequency of the genetic variation between the healthy controls and the PD patients (OR = 1.52, 95%CI: 0.15–8.15, *p* = 0.64). Between the definite iNPH patients and PD patients, there was a significant difference in the frequency of the genetic variation (OR = 5.18, 95%CI: 1.05–50.84, *p* = 0.038) (Fig 2).

Finally, copy number gain of the *SFMBT1* gene was analyzed using Fisher’s exact test. The results showed that there were no significant differences between iNPH and control (p = 0.060), between iNPH and PD (p = 1), and between PD and control (p = 0.063).

## Discussion

The present study examined the *SFMBT1* gene in patients with shunt-responsive definite iNPH, healthy elderly and PD patients, and found that a segmental copy number loss in intron 2 of the gene was more frequently observed in the iNPH patients than in the healthy elderly and PD patients. Therefore, the alteration in the gene may be a genetic risk for definite iNPH.

NPH is usually classified as idiopathic NPH (iNPH) or secondary NPH after subarachnoid hemorrhage, meningitis, head trauma, and so on. Recently, a third form of NPH, familial NPH (fNPH), has widely been recognized [4–8]. Clinical and brain MRI features of fNPH are indistinguishable from those of iNPH, suggesting that genetic factor(s) may also be involved in the pathogenesis of sporadic iNPH [4–8]. This speculation is supported by a study that showed an increased frequency of two or more symptoms of iNPH triads was observed among first-degree relatives of sporadic iNPH patients [7]. However, little is known about the genetic risk for iNPH. The increased allele frequency of apolipoprotein E epsilon4 (ApoE4) was reported to be a possible genetic risk for iNPH in a small number of iNPH samples (n = 13) [17]. Later, in another study (n = 15), the ApoE3/3 genotype was shown to be more frequently observed in shunt-responsive iNPH patients as compared with other genotypes [18]. However, in a recent study using more samples of iNPH, ApoE genotypes have been shown to distribute similarly in shunt-responsive (n = 94) and non-responsive (n = 16) iNPH patients and healthy controls [19].

In our previous study, we reported that a segmental copy number loss of intron 2 of the *SFMBT1* gene was frequently observed in subjects with possible iNPH and AVIM [9]. The copy number loss was heterozygous and occurred at the 12 kb region within intron 2 of the *SFMBT1* gene [9]. In that study, however, the number of subjects examined was very small (n = 8); therefore, it was not proper to perform a statistical analysis. Moreover, subjects were possible iNPH and AVIM but not shunt-responsive definite iNPH. In the present study, we examined many patients with shunt-responsive definite iNPH, and our statistical analysis demonstrated that the frequency of the copy number loss in intron 2 of the *SFMBT1* gene was significantly higher in definite iNPH patients than in healthy subjects. The results were replicated in two independent populations of iNPH patients and controls. Furthermore, the statistical significance was maintained even when PD patients were used as the disease control. Thus, the genetic change in the *SFMBT1* gene seems to be a genetic risk for definite iNPH.

In the present study, 26% of the iNPH patients had a copy number loss in intron 2 of the gene. One may speculate that it may possibly represent a hereditary disorder. Previously, we reported a large family with NPH with an autosomal-dominant inheritance [6]. In the present study, we collected samples from sporadic iNPH patients with no family history. Therefore, it seems unlikely that a subset of the patients we collected is a hereditary disorder with a Mendelian inheritance. Alternatively, as other multi-factorial diseases which are caused by a combination of genetic, environmental and lifestyle factors, such as diabetes mellitus and cerebrovascular disease, it seems reasonable to consider that the *SFMBT1* gene alteration would be a genetic predisposition factor of several risk factors for iNPH.

Using the human HapMap panel, it has been reported that the frequencies of copy number loss in intron 2 of the gene were 0.37% (1/270) [20], 9.8% (11/112) [21], and 11.9% (15/126) [22]. The frequencies in a mixed population of Han Chinese (CHB) and Japanese (JPT) were described as 6.7% (6/90) and 7.8% (7/90), respectively [23,24]. In the present study, the frequency of copy number loss in intron 2 of the gene was 4.2% in control subjects, which is similar to the frequency reported.

The *SFMBT1* gene is localized on the region of chromosome 3p21.1 in the human. The gene shares a high similarity with the *Drosophila Scm* (*sex comb on midleg*) gene. The *SFMBT1* gene encodes a protein with 866 amino acid residues, which contains four malignant brain tumor (MBT) repeat domains [25,26]. SFMBT1 mRNA has been reportedly expressed in many cells and tissues, including the brain [27]. In the brain, SFMBT1 protein was localized in the epithelial cells of the choroid plexus, the ependymal cells lining the ventricles, and the endothelial and muscle cells of the blood vessels [9], all important structures for secretion, flow, and absorption of CSF. Therefore, it seems that the genetic change in the *SFMBT1* gene may influence the circulation of CSF in the brain. With regard to a functional significance of the copy number loss, it is noteworthy that the binding site of BAF155 (Brg-1-Associated Factor, 155 kD) is present within the region of copy number loss in intron 2 of the gene [28]. Therefore, it is possible that the deletion of the region containing the binding site in intron 2 may influence a function of the gene. Alternatively, the copy number loss may be just a non-functional polymorphism in linkage disequilibrium with other genetic variations of functional importance in the vicinity. Further studies are needed to clarify the mechanism by which the copy number loss in intron 2 of the gene confers increased risk of iNPH.

## Conclusion

The present study demonstrates that a copy number loss in intron 2 of the *SFMBT1* gene may be a genetic risk for shunt-responsive definite iNPH. Further studies of SFMBT1 will contribute to the elucidation of the molecular basis of iNPH.

## Supporting Information

S1 Fig Deletions in intron 2 of the SFMBT1 gene (Database of Genomic Variants). A large deletion containing the binding site of the reference probe (Hs06629891_cn) is found only in one (0.09%) person among the 1109 persons examined. Most deletions in this region do not affect it.
(TIF)

S2 Fig PCR-based amplification of the region (14.8 kbp) with or without deletion (loss) in intron 2 of the *SFMBT1* gene. A single fragment of 14.8 kbp was obtained by PCR amplification from template DNA without deletion in intron 2. On the other hand, two fragments, 14.8 kbp and 3.8 kbp bands, were obtained from the template DNA with the deletion in intron 2, indicating that approximately 11 kbp loss (deletion) is present in this region. The primers used are as follows Forward: 5'-CACCCAGTCCAACAGTCCTC-3' Reverse: 5'-CCTCCTCATCCTTCCTCCC-3'
(TIF)
