# Supplementary figures and images for: A Segmental Copy Number Loss of the SFMBT1 Gene Is a Genetic Risk for Shunt-Responsive, Idiopathic Normal Pressure Hydrocephalus (iNPH): A Case-Control Study
Source: PLoS One. 2016 Nov 18;11(11):e0166615. doi: 10.1371/journal.pone.0166615 (PMC5115754; doi:10.1371/journal.pone.0166615)

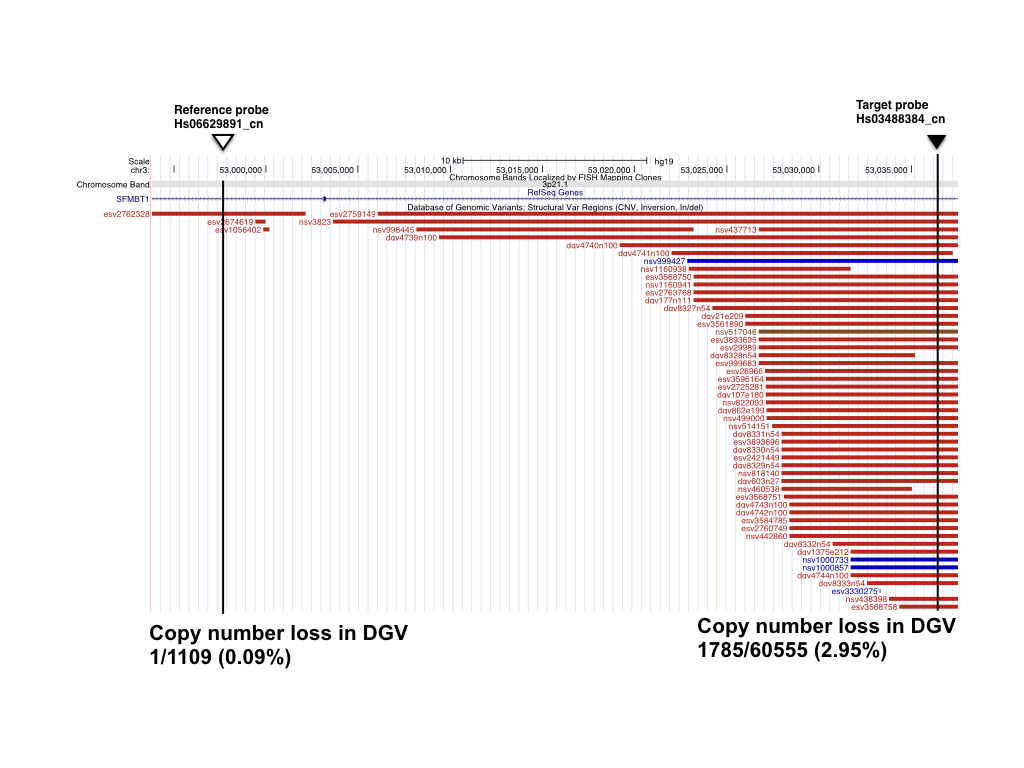

Supplement: S1 Fig — A large deletion containing the binding site of the reference probe (Hs06629891_cn) is found only in one (0.09%) person among the 1109 persons examined. Most deletions in this region do not affect it. (TIF) [file pone.0166615.s001.tif]

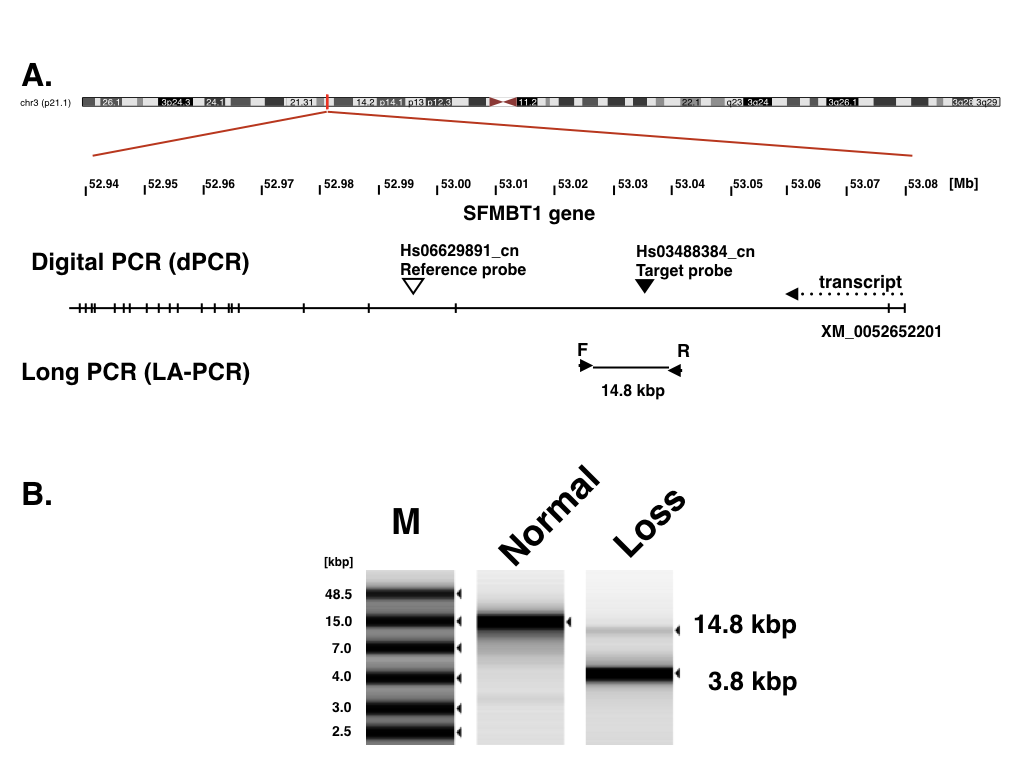

Supplement: S2 Fig — A single fragment of 14.8 kbp was obtained by PCR amplification from template DNA without deletion in intron 2. On the other hand, two fragments, 14.8 kbp and 3.8 kbp bands, were obtained from the template DNA with the deletion in intron 2, indicating that approximately 11 kbp loss (deletion) is present in this region. The primers used are as follows Forward: 5'-CACCCAGTCCAACAGTCCTC-3' Reverse: 5'-CCTCCTCATCCTTCCTCCC-3' (TIF) [file pone.0166615.s002.tif]
